# Supplementary figures and images for: Genetic architecture of gene expression traits across diverse populations
Source: PLoS Genet. 2018 Aug 10;14(8):e1007586. doi: 10.1371/journal.pgen.1007586 (PMC6105030; doi:10.1371/journal.pgen.1007586)

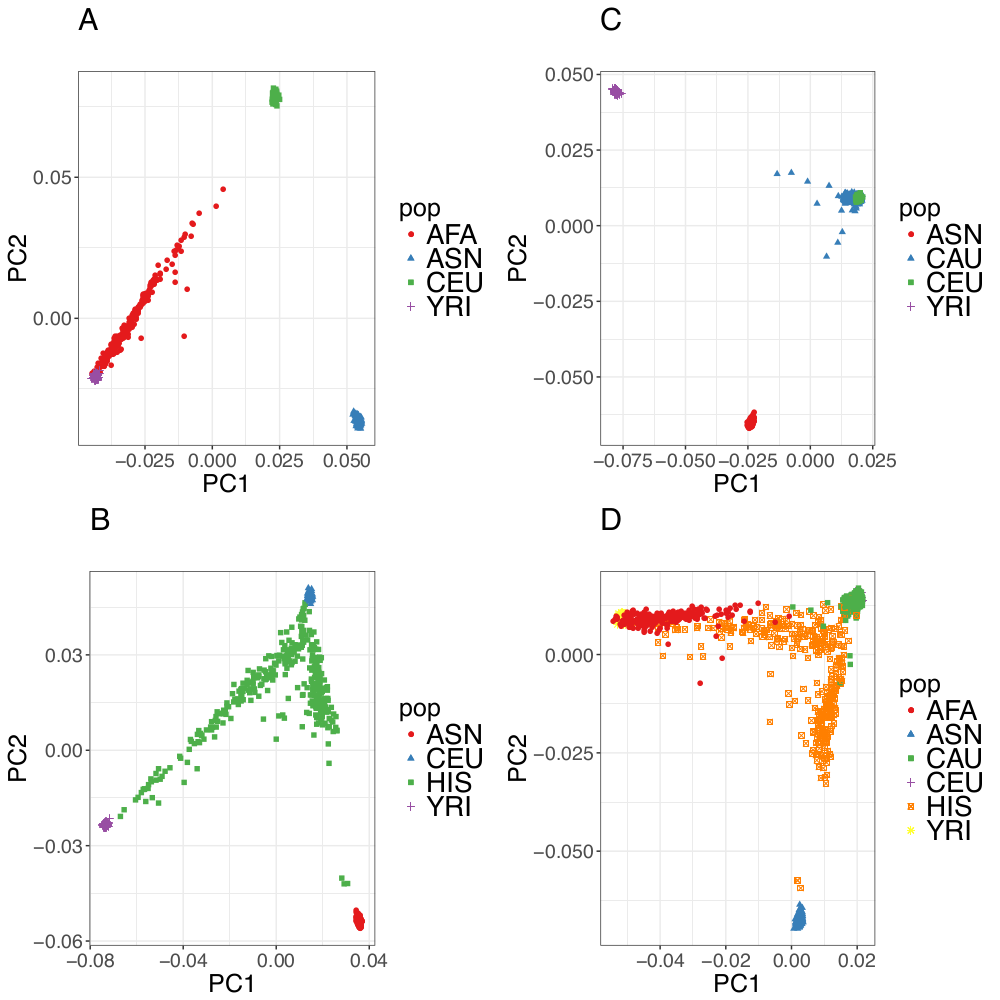

Supplement: S1 Fig — PC1 vs. PC2 plots of each MESA population when analyzed with HapMap populations show varying degrees of admixture. The HapMap populations are defined by the following abbreviations: Yoruba from Ibadan, Nigeria (YRI), European ancestry from Utah (CEU), East Asians from Beijing, China and Tokyo, Japan (ASN). (A) MESA AFA population (red), (B) MESA HIS population (green), (C) MESA CAU population (blue), (D) all MESA populations combined. (TIF) [file pgen.1007586.s001.tif]

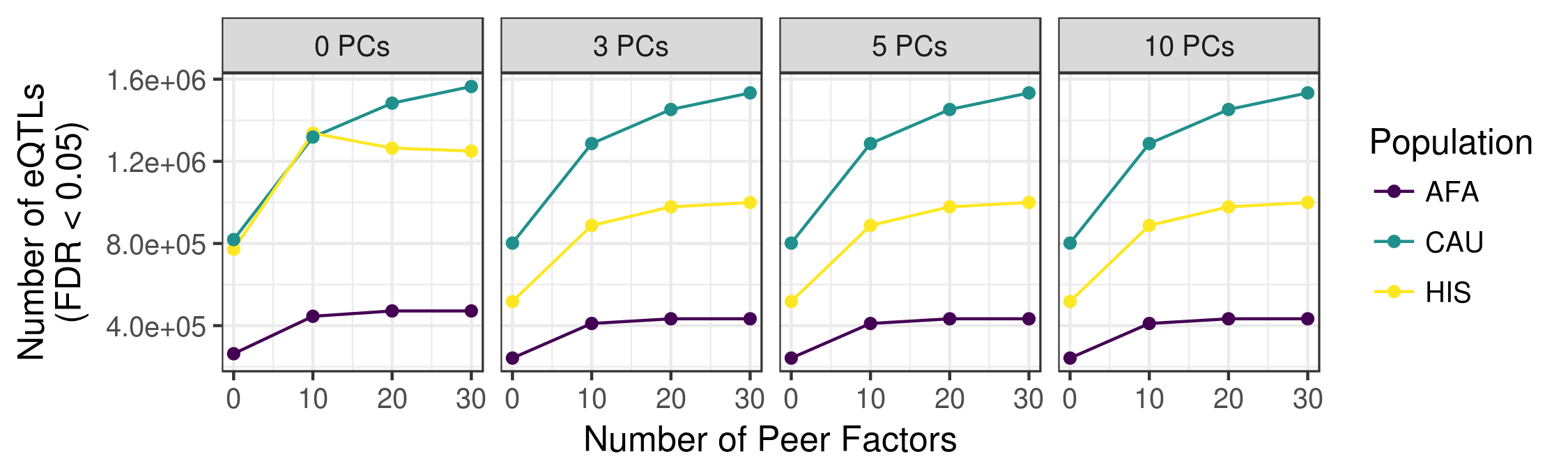

Supplement: S2 Fig — cis-eQTL count (FDR < 0.05) vs. the number of PEER factors used to adjust for hidden confounders in the expression data of each MESA population. The number of genotypic PCs is listed in the gray title box and the color of the lines represent each MESA population. Note that all curves with at least 3 genotypic PCs look the same. AFA = MESA African American, CAU = MESA European American, HIS = MESA Hispanic American. (TIFF) [file pgen.1007586.s002.tiff]

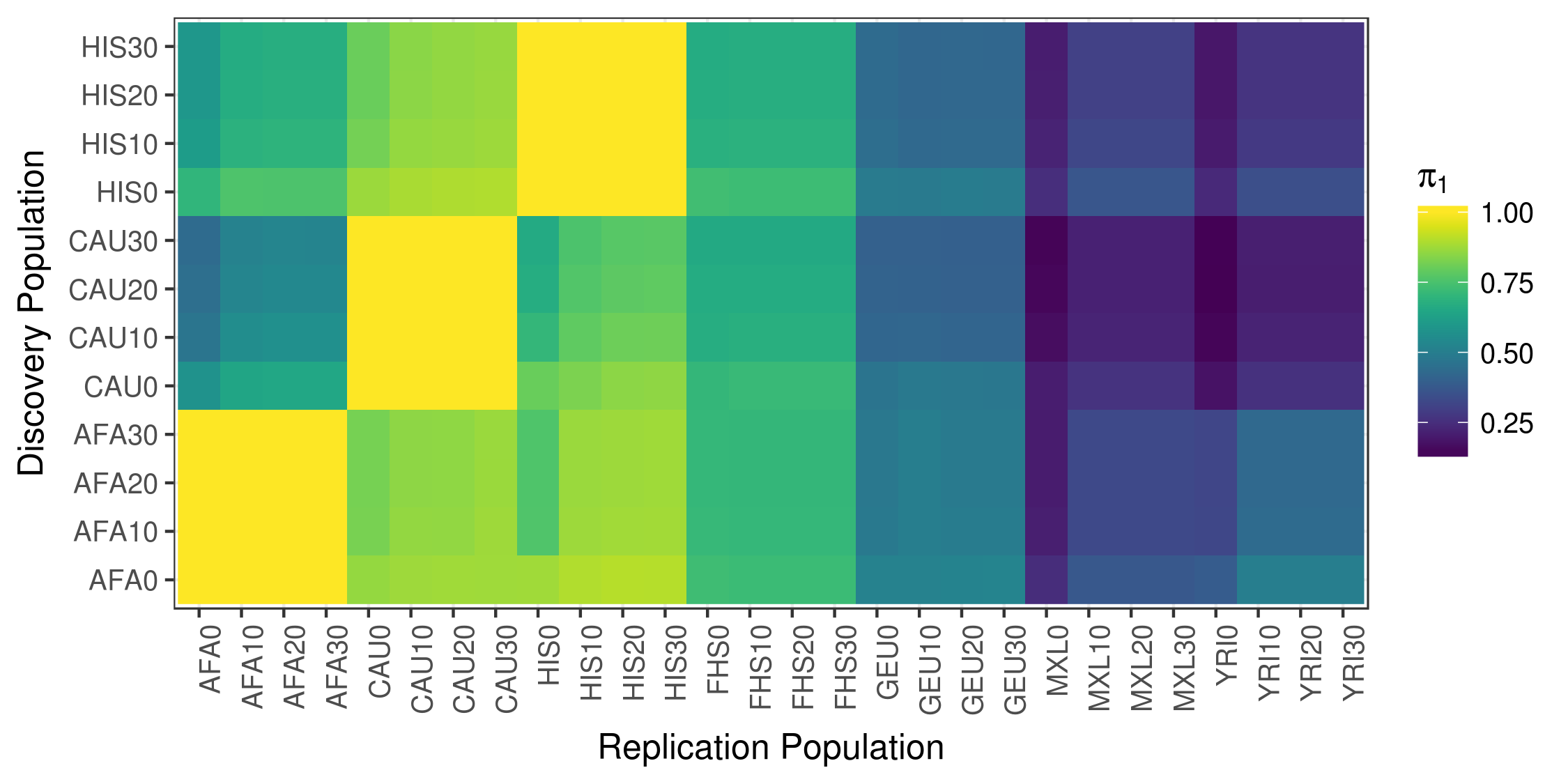

Supplement: S3 Fig — True positive rate π1 statistics [29] for cis-eQTLs are plotted comparing each Discovery Population to each Replication Population. The number after each population abbreviation is the number of PEER factors used to adjust for hidden confounders in the expression data. Higher π1 values indicate a stronger replication signal. π1 is calculated when the SNP-gene pair from the discovery population is present in the replication population. All models shown included 3 genotypic principal components. AFA = MESA African American, CAU = MESA European American, HIS = MESA Hispanic American, FHS = Framingham Heart Study, GEU = Geuvadis, MXL = Mexicans in Los Angeles, YRI = Yoruba in Ibadan, Nigeria. (TIFF) [file pgen.1007586.s003.tiff]

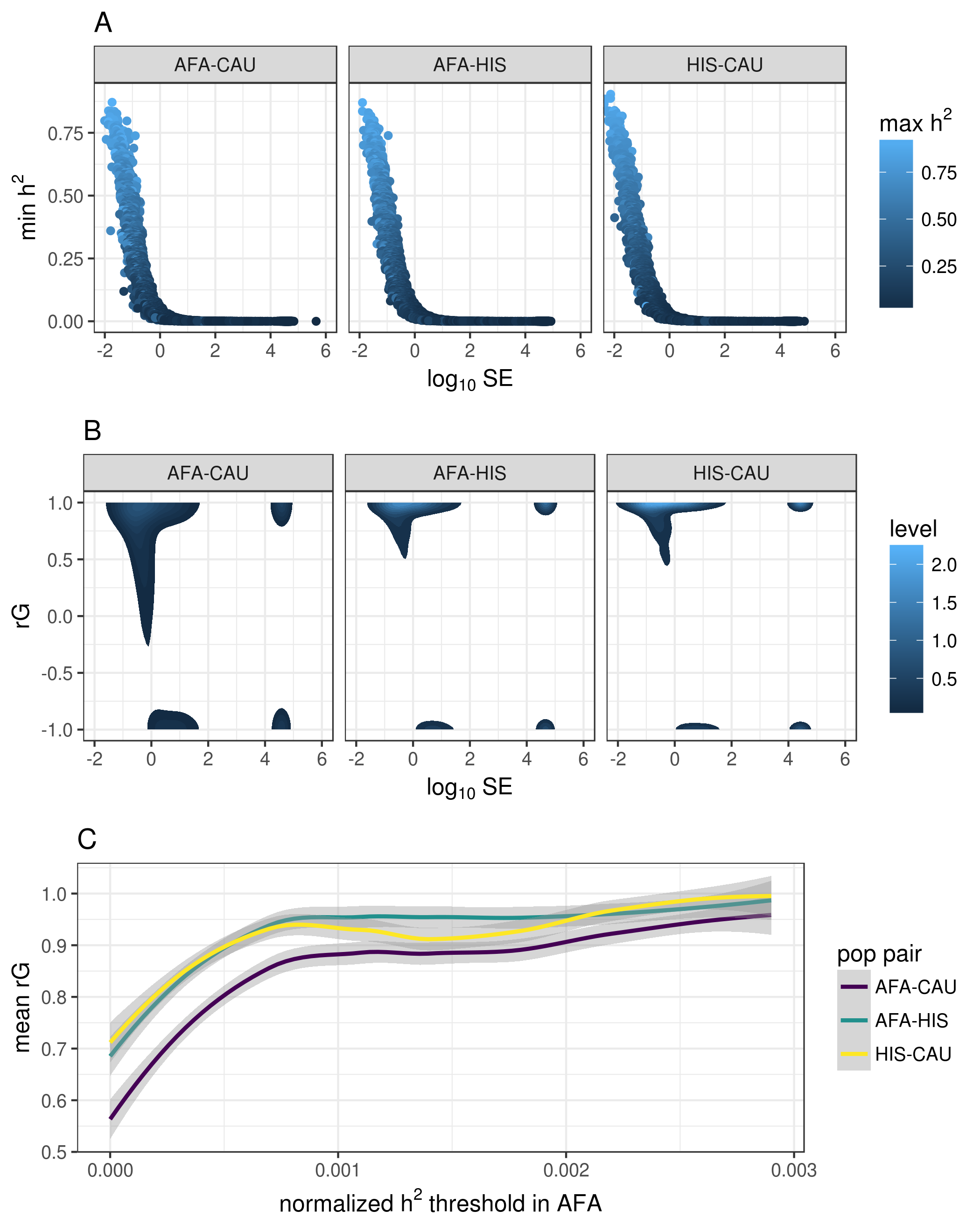

Supplement: S4 Fig — (A) Pairwise population comparison of minimum heritability (h2) and rG standard error (SE) for each gene. The y-axis is the minimum h2, the x-axis is the −log10 SE of the rG estimate, and the points are colored according to the maximum h2 between the populations titling each plot. (B) rG compared to −log10 SE of the estimate. Genes with low SE are more likely to have a positive rG estimate. (C) Comparison of the genetic correlation between pairwise MESA populations and the subset of genes with normalized h2 greater than a given threshold in the AFA population. h2 estimates are normalized by the number of SNPs used in the estimate, i.e. those within 1 Mb of each gene. (TIFF) [file pgen.1007586.s004.tiff]

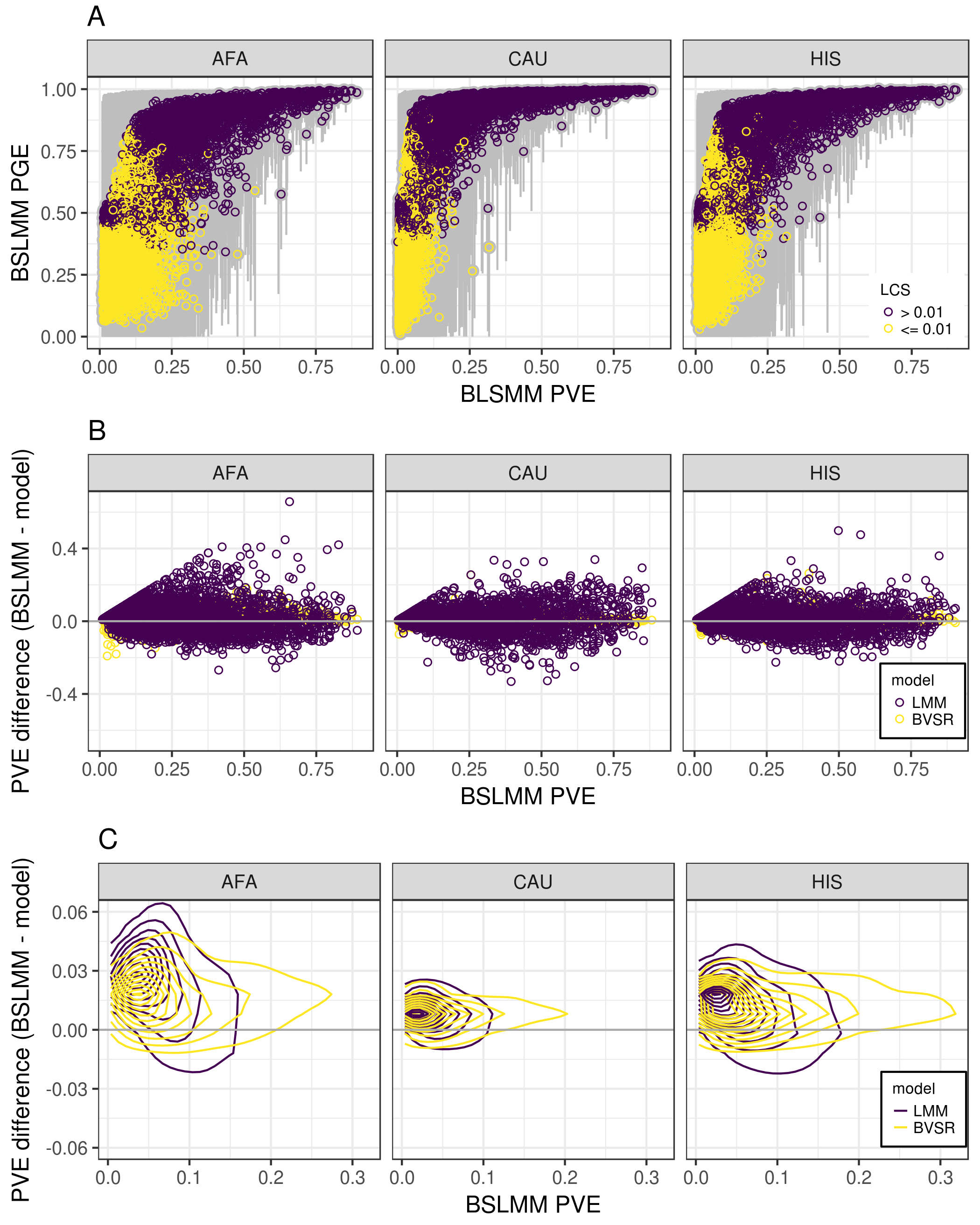

Supplement: S5 Fig — (A) Bayesian Sparse Linear Mixed Modeling (BSLMM) includes both sparse and polygenic components and estimates the total percent variance explained (PVE) and the parameter PGE, which represents the proportion of the genetic variance explained by sparse effects. The highly heritable genes (high PVE) have PGE near 1 and therefore the local genetic architecture is sparse. There is not enough evidence to determine if the lower heritablility genes are more sparse or polygenic. (B) The difference between PVE of BSLMM and LMM or BVSR is compared to the BSLMM PVE across genes in MESA populations AFA, HIS, and CAU. (C) Zoomed in plot of B using contour lines from two-dimensional kernel density estimation to visualize where the points are concentrated. For both LMM and BVSR, the PVE difference values (y-axis) are above the horizontal line at zero indicating that both models perform worse than BSLMM. However, the difference between LMM and BSLMM is greater than between BVSR and BSLMM, which indicates sparse effects predominate for most genes. (TIFF) [file pgen.1007586.s005.tiff]

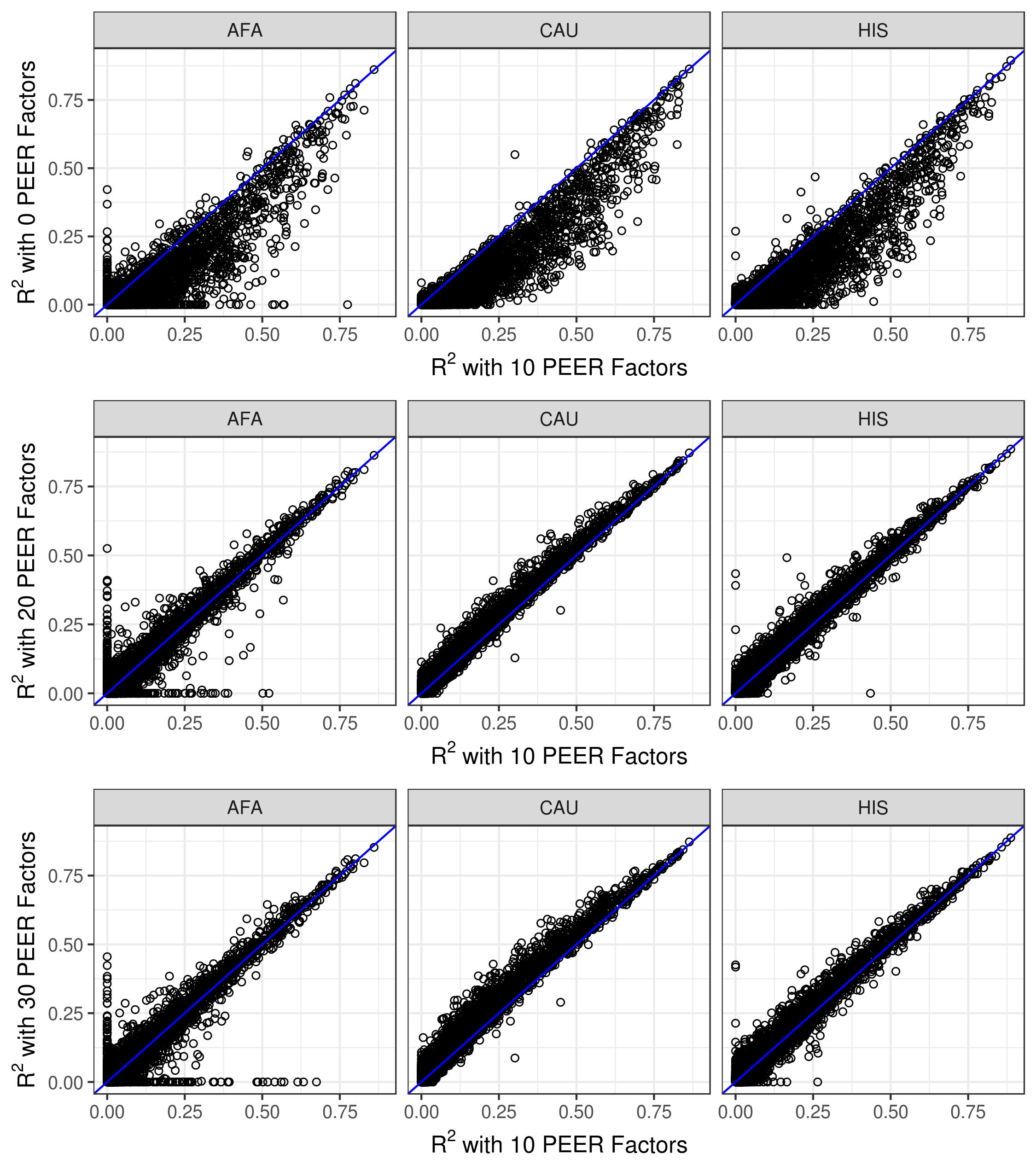

Supplement: S6 Fig — Comparison of the elastic net (α = 0.5) cross-validated predictive performance R2 in models with different numbers of PEER factors as covariates. Across populations, models with 10 PEER factors shows increased predictive performance over 0 PEER factors, while models with 10, 20, or 30 PEER factors perform similarly. (TIFF) [file pgen.1007586.s006.tiff]

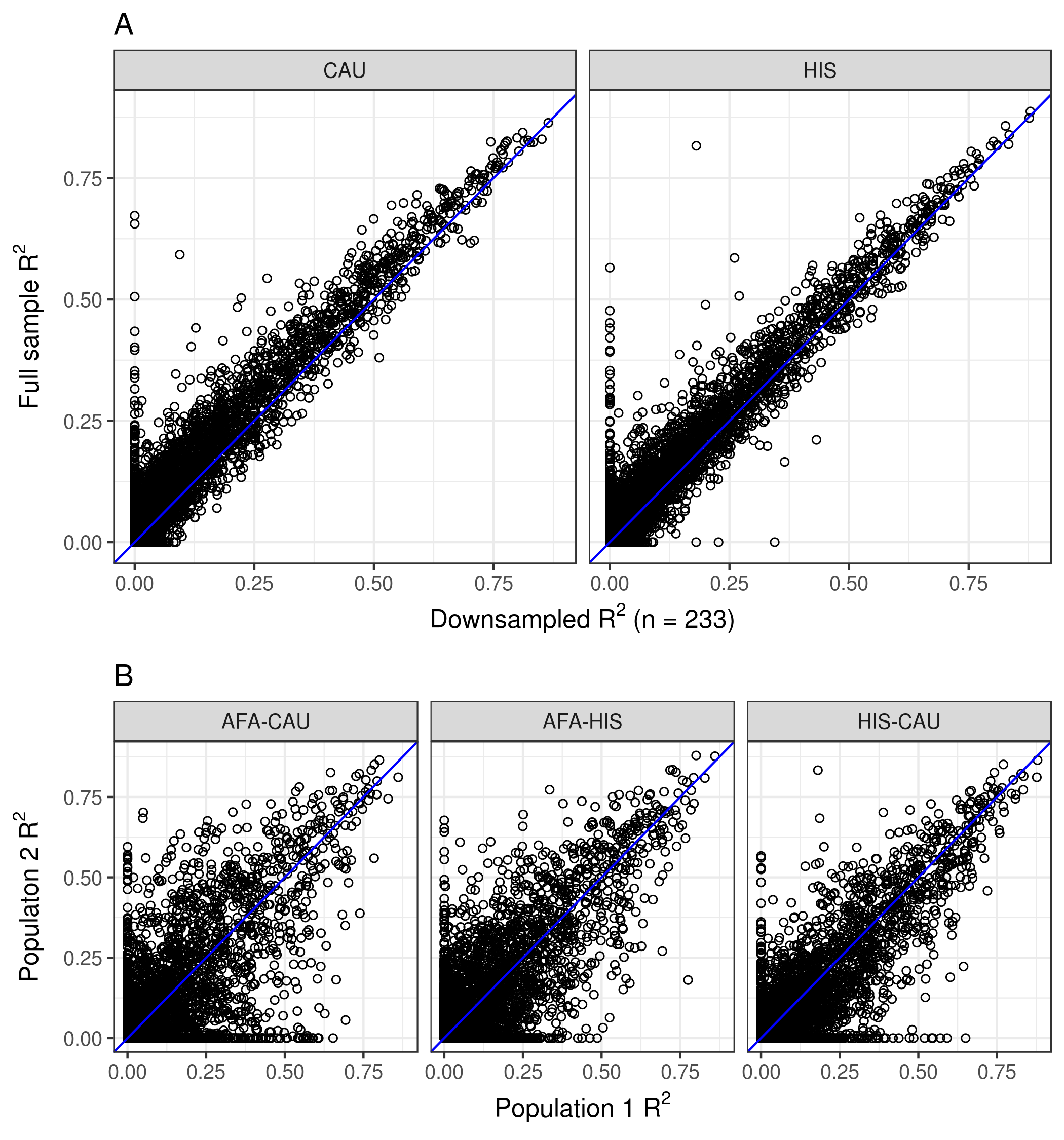

Supplement: S7 Fig — The CAU and HIS populations were randomly downsampled to include the same sample size as AFA (n = 233). Predictive performance was measured within each population using nested cross-validation. (A) Comparison of the elastic net (α = 0.5) predictive performance R2 of the full sample to the downsampled population. Spearman correlations were 0.81 and 0.83 for CAU and HIS sample comparisons, respectively. (B) Comparison of predictive performance for each gene (R2) between each pair of populations. In each gray title box, population 1 is listed first and population 2 is listed second. The identity line is shown in blue. The pairwise Spearman correlations (ρ) between genes are AFA-CAU downsample: ρ = 0.54, AFA-HIS downsample: ρ = 0.61, HIS downsample-CAU downsample: ρ = 0.65. (TIFF) [file pgen.1007586.s007.tiff]

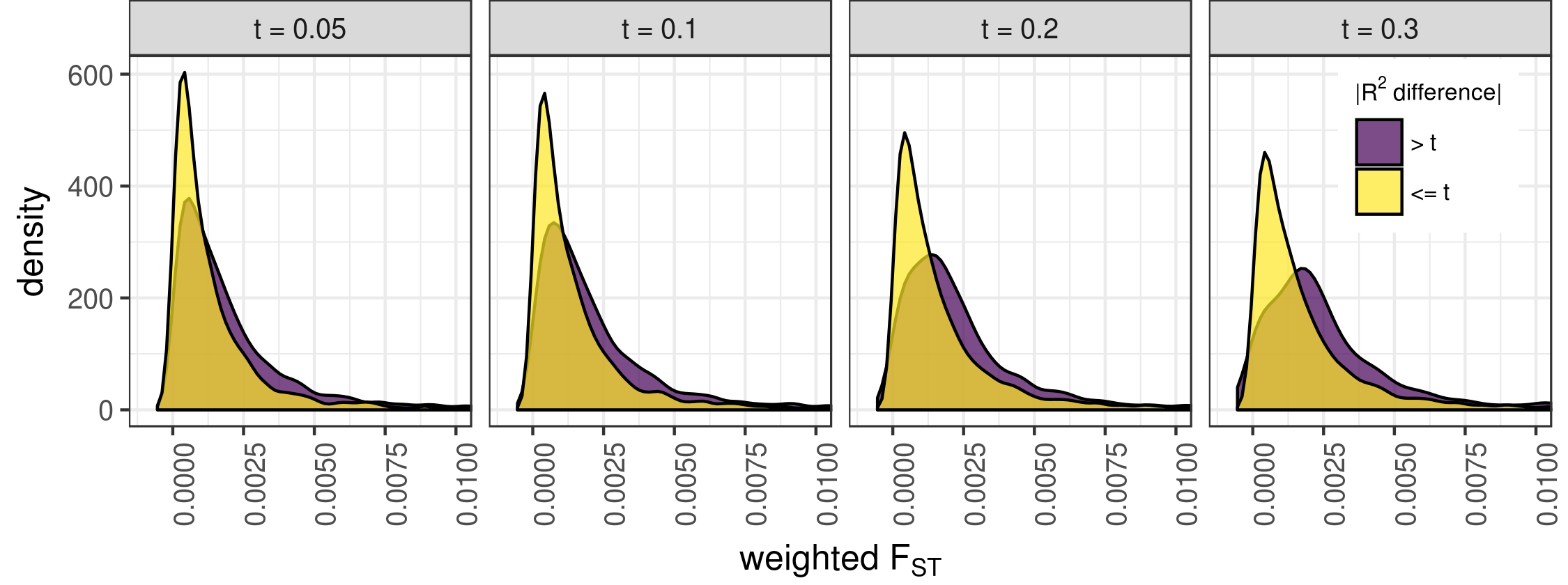

Supplement: S8 Fig — For each gene model, weighted average FST was calculated by multiplying each beta from the elastic net model by that SNP’s FST before taking the mean across SNPs. The gene groups with the larger absolute value R2 difference between populations had significantly larger weighted FST at each difference threshold, t (Wilcoxon rank sum tests, P < 2.2 ×10^−16^). (TIFF) [file pgen.1007586.s008.tiff]
